# Kinematics Modeling and Simulation of a Bionic Fish Tail System Based on Linear Hypocycloid

**DOI:** 10.1155/2015/697140

**Published:** 2015-08-19

**Authors:** Shu-yan Wang, Jun Zhu, Xin-guo Wang, Qin-feng Li, Hui-yun Zhu

**Affiliations:** School of Mechanical Engineering, Jiangsu University of Science and Technology, Zhenjiang 212203, China

## Abstract

Kinematics and simulation study on a two-joint linear hypocycloid tail driving system composed of a special planetary gear system and a linkage mechanism are conducted in this paper. First, the composition and working principle of the linear hypocycloid tail transmission system are introduced and analyzed. Second, the kinematics study on the transmission mechanism is conducted with graphical method of vector equation. The relationships between the caudal peduncle stroke, the tail fin swing angle, and the phase difference with structure parameters are studied, and further optimization of structure sizes (i.e., linkage length, sun gear's diameter, the intersection angle between planet gears, etc.) is developed. At last, simulation and comparative study on a biofish in sample parameters with a live fish of Carp is conducted in MATLAB. The study would serve for underwater vehicles thruster design and its mechanism.

## 1. Introduction

Bionic propulsion device inspired by fish swimming skills to replace traditional underwater devices has caught much attention of biologists and engineers all over the world. Compared to traditional screw propellers, bionic fish propulsion has its unique advantages in high efficiency, low noise, and great mobility [1–3]. Previous investigations have shown that fish swimming in BCF mode can obtain a larger propulsion force during escape and prey, but fish swimming in MPF mode can obtain higher stability and maneuverability [4]. 85% of fish are swimming in BCF mode for power supply, supplemented by MPF mode to keep bodies balance, retreating, hovering, and turning movement [1].

Some researchers devote themselves to reveal kinematics and hydrodynamics of live fish. Lightill [5] put forward the elongated-body theory where the movements of any horizontal section of caudal fin, with yaw angle fluctuating in phase with its velocity of lateral translation, were studied for different positions of the yawing axis. In addition, the proposed theory was extended to the large-amplitude elongated-body theory so that a prediction of instantaneous reactive force between fish and water was achieved for fish motions of arbitrary amplitude [6]. Vo et al. [7] proposed an analytical optimization approach which can guarantee the maximum propulsive velocity of fish robot in the given parametric conditions. Other researchers proposed various bionic vehicles to mimic the swimming of live fish. Morgansen et al. [8] designed a planar carangiform robot fish with motion control algorithms to obtain experimental trajectory tracking results. Esposito et al. [9] presented a robotic fish caudal fin with six individually moveable fin rays based on sunfish tail. Fay et al. [10] developed a wireless aquatic bionic fish with a wireless video camera, a controller, and polypyrrole actuators to detect and analyze pollutants in natural waters. Yun et al. [11] applied a special waving caudal fin with vertical phase differences to reduce reaction torque and to improve bionic fish's velocity and stability.

The purpose of this paper is to design a linear hypocycloid driving mechanism, which has advantages of combining speed reducer with transformation mechanism, and adjustable phase difference between caudal peduncle and tail fin. In this paper, kinematics of the driving system was analyzed comprehensively, and structural parameters optimization is developed for mimicking a real fish tail's oscillating motion, which is verified by a further comparative study with a live Carp.

## 2. Structure and Working Principle

**Figure 1:**
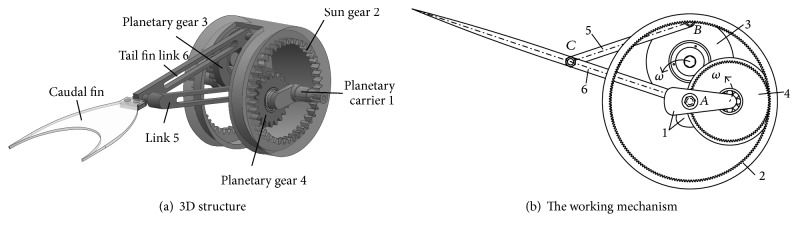
The bionic tail driving system.

The bionic fish tail system based on linear hypocycloid is composed of a special planetary gear train with the reference circle of the planetary gear whose reference radius is half of the sun gear and a plane linkage which can form a variable triangle motion relation, as shown in Figure 1(a). The proposed special planetary gear train involves a *V* type planetary carrier, two planetary gears installed at the *V* planetary carrier in two parallel planes, and the sun gear. The plane linkage consists of two links: one end of two links is connected to rotate with a fixed point located at the reference circle of a planetary gear, respectively, and the other end of two links is connected to rotate at a certain point.

The working mechanism of the bionic fish tail system based on linear hypocycloid is shown in Figure 1(b). When the two planetary gears 3 and 4 meshed with the sun gear 2 under the driving of the *V* planetary carrier 1 at a certain speed, Point *A* or Point *B* located at the reference circle of the planetary gear 4 or 3 will be reciprocating along the connected line of the point and the sun gear's central point. In case of Point *A* and Point *B* designed at one same diameter line of the sun gear 2, both Point *A* and Point *B* would be reciprocating in the diameter line *AB* with a phase difference decided by *V* shape's angle of *V* planetary carrier 1. With the reciprocating motion of Point *A* and Point *B* with a certain phase difference, a motion triangle *ABC* in the plane linkage mechanism is formed. As a result, the tail fin link 6 will rotate around Point *A* and make reciprocating movement with Point *A* along the diameter line *AB*. Obviously, the relative length between *AC* and *BC*, the length of *AC* or *BC*, *V* shape's angle of *V* planetary carrier, and the diameter of the sun gear will be important parameters for the driving system, which will be further discussed in this paper.

## 3. Kinematics on the Tail Driving System

### 3.1. Foundation of Coordinate System

**Figure 2:**
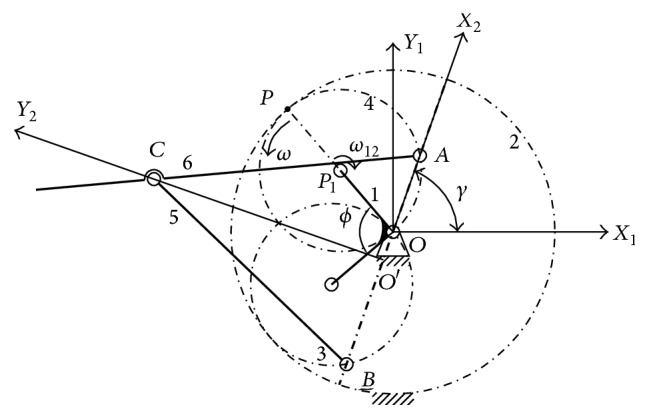
The working principle of the tail driving system.

Three coordinate systems are employed for the tail driving system as shown in Figure 2. The first fixed coordinate system is *O*-*X*_1_ *OY*_1_ with the centre of the sun gear 2 as the origin Point *O*, *X*_1_ axis points at the horizontal direction, and *Y*_1_ axis is upward. With the planetary gear 4 meshing with the sun gear, the second moving coordinate system *O*′-*X*_2_ *O*′*Y*_2_ is connected to the connection line of Point *A* and Point *B*, the origin point *O*′ is central point of the sun gear, *O*′*X*_2_ is along the line of *AB*, and *O*′*Y*_2_ is perpendicular to the line of *AB*.

### 3.2. Kinematics of the Tail Driving System

#### 3.2.1. Kinematics of the Planetary Gear Train

Kinematic relation of the linear hypocycloid planetary gear train can be deduced easily based on relative kinematics. When the planetary gear meshed with the sun gear at pitch Point *P* shown in Figure 2, in *X*_1_ *OY*_1_ coordinate system, velocity vector equation could be written as

(1) $$\begin{array}{c} {\vec{V}}_{P_{1}O}+{\vec{V}}_{PP_{1}}={\vec{V}}_{P}. \end{array}$$

Here, ${\vec{V}}_{P_{1}O}$ is the relative velocity of Point *P*_1_ to Point *O*, ${\vec{V}}_{PP_{1}}$ is the relative velocity of Point *P* to Point *P*_1_, ${\vec{V}}_{P}$ is the absolute velocity of the planetary gear 4 at the meshing Point *P*. When the sun gear is fixed, ${\vec{V}}_{P}=0$. Then (1) could be replaced as

(2) $$\begin{array}{c} OP_{1}\omega +P_{1}P\omega_{12}=0. \end{array}$$

Here, *ω* is the angular velocity of planetary gear 4 in its revolution, and *ω*_12_ is the angular velocity of planetary gear 4 in its rotating motion. *OP*_1_ = *P*_1_ *P* = *R*, and *R* is the reference radius of planetary gear 4.

Therefore, *ω* = −*ω*_12_, which shows *ω* and *ω*_12_ with the same magnitude but in opposite direction, as shown in Figure 2.

#### 3.2.2. The Working Mechanism of Linear Hypocycloid

**Figure 3:**
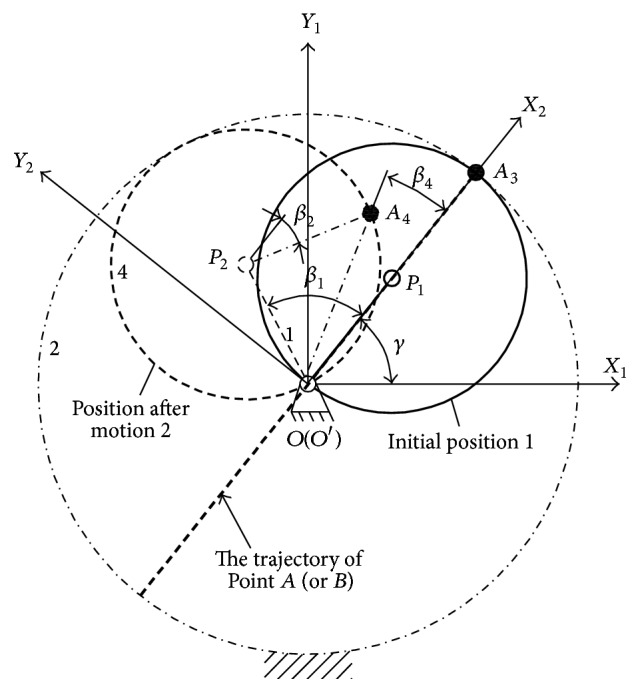
The working mechanism of linear hypocycloid.

For the proposed hypocycloidal gear train in this paper, the track of arbitrary point located at the reference circle of planetary gear should be one certain diameter line of the sun gear. The specific working mechanism of linear hypocycloid is shown in Figure 3. In the moving coordinate system *X*_2_ *O*′*Y*_2_, an arbitrary point located at the reference circle of planetary gear 4 is marked as Point *A*_3_, and the position of *A*_3_ would be supposed to move to a new spot marked with *A*_4_ after planetary motion with any time *t*. Based on the closed vector triangle **O**′**P**_2_ **A**_4_, equations would be gained as follows:

(3) OP2sin⁡β1+P2A4sin⁡β2=OA4sin⁡β4,OP2cos⁡β1+P2A4cos⁡β2=OA4cos⁡β4.

Here, *β*_1_ is the angle of *OP*_1_ and *OP*_2_, and *β*_2_ is the angle between *P*_2_ *A*_4_ and *OX*_2_. *OP*_2_ = *P*_2_ *A*_4_ = *R* = *D*/4, where *D* is the reference diameter of the sun gear 2, and *β*_1_ = −*β*_2_ with the proposed relation *ω* = −*ω*_12_.

As a result, *β*_4_ must be equal to zero, so (4) could be simplified as

(4) $$\begin{array}{c} S_{A}=\frac{D}{2}\cos\left(\;\omega t\;\right). \end{array}$$

Equation (4) shows that Point *A* is reciprocating along the diameter line in harmonic motion, and its stroke is the diameter *D* of the sun gear.

If Points *A* and *B* were selected at the same diameter line of the sun gear but in different planetary gears installed at *V* planetary carrier, Point *B* should also do reciprocating motion along the same diameter line of sun gear with a certain phase difference *ϕ*, and the phase difference *ϕ* is decided by *V* shape's angle of the planetary carrier. Therefore, the motion equation *S*_*B*_ of Point *B* could be described as

(5) $$\begin{array}{c} S_{B}=\frac{D}{2}\cos\left(\;\omega t+\phi \;\right). \end{array}$$

The proposed *S*_*A*_ and *S*_*B*_ were deduced in the moving coordinate system *X*_2_ *O*′*Y*_2_. If putting *S*_*A*_ and *S*_*B*_ into the fix coordinate system *X*_1_ *OY*_1_, the equations of Point *A* and Point *B* could be deduced as

(6) XSAYSA=D2cos⁡ωt·cos⁡γsin⁡γ,XSBYSB=D2cos⁡ωt+ϕ·cos⁡γsin⁡γ.

Here, *γ* is the angle between the *OX*_1_ axis and the *O*′*X*_2_ axis.

#### 3.2.3. The Working Mechanism of the Motion Triangle

**Figure 4:**
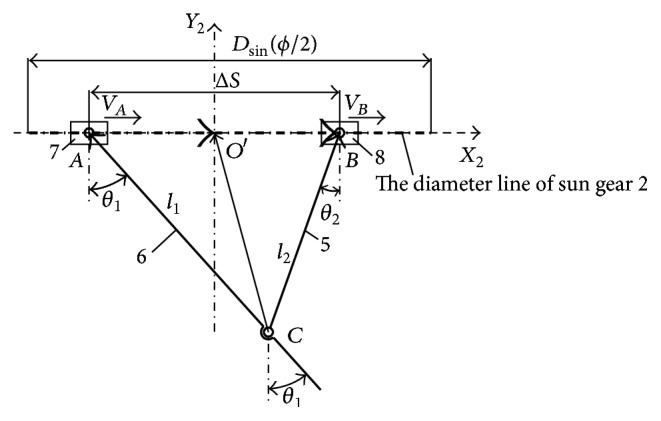
The working mechanism of the equivalent mechanism.

To simplify kinematic analysis, the link mechanism in the driving system would be replaced with an equivalent mechanism by removing the planetary gear train, shown in Figure 4. In the equivalent mechanism, link 5, tail fin link 6, slider 7, and slider 8 are connected at Points *A*, *B*, and *C* to form the equivalent mechanism. Here, link 5 would rotate around Point *B* of slider 8, the tail fin link 6 would rotate around Point *A* of slider 7, and the tail fin link 6 and link 5 are connected to rotate at Point *C*. Slider 7 and slider 8 do the same reciprocating motion with Points *A* and *B*, respectively, so the motion triangle *ABC* still remained the same as the driving system. As a result, the tail fin link 6 would gain a composite motion of reciprocating with slider 7 along line *AB* and oscillating around Point *A*.

Based on vector triangles **A** **B** **C** and **A** **O**′**C**, kinematics model of the equivalent mechanism would be established as

(7) l1cos⁡θ1−l2cos⁡θ2=0,l1sin⁡θ1−l2sin⁡θ2=ΔS,SA−l1sin⁡θ1=Scx,−l1cos⁡θ1=Scy.

Here, *θ*_1_ is the swing angle of the tail fin link 6, *θ*_2_ is the swing angle of link 5, *l*_1_ and *l*_2_ are rod lengths of the tail fin link 6 and link 5, respectively, *S*_*A*_ is the motion position of slider 7, *S*_*cx*_ is the motion position of Point *C* in *O*′*X*_2_ axis, and *S*_*cy*_ is the motion position of Point *C* in *O*′*Y*_2_ axis.

The instantaneous position distance Δ*S* of the two sliders could be described as

(8) $$\begin{array}{c} \Delta S=S_{B}-S_{A}=\frac{D}{2}\sin\left(\;\frac{\phi }{2}\;\right)\sin\left(\;\omega t+\frac{\phi }{2}\;\right). \end{array}$$

Based on (7) and (8), displacement equations of the tail fin link 6 with composite motion of reciprocating and oscillating could be described as

(9) θ1=arcsin⁡ΔS2+l12−l222l1·ΔS,Scx=SA−ΔS2+l12−l222ΔS.

With derivation of (9), velocity equations of the tail fin link could be deduced as

(10) ω1=ΔVl1cos⁡θ1−ΔVtan⁡θ1ΔS,vcx=vA−l1ω1cos⁡θ1.

Here, *ω*_1_ is the angular velocity of the tail fin link 6, *v*_*A*_ is the velocity of slider 7, and Δ*V* is velocity difference between slider 7 and slider 8.

With derivation of (10), acceleration equations of the tail fin link could be deduced as

(11) α1=2ω1ΔVΔS+ω12l1−ΔaΔStan⁡θ1+ΔV2ΔS+Δa·1l1cos⁡θ1,acx=aA+l1ω12sin⁡θ1−l1α1cos⁡θ1.

Here, *α*_1_ is the angular accelerated velocity of the tail fin link 6, *a*_*cx*_ is the accelerated velocity of Point *C* along *O*′*X*_2_, *a*_*A*_ is the accelerated velocity of slider 7, and Δ*a* is the accelerated velocities difference of two sliders.

## 4. Optimal Design on Structural Parameters

It is obvious that specific parameters such as the phase difference, rod length, relative length of two rods, and reference diameter of the sun gear will directly or indirectly affect the behavior of tail link 6. In this chapter, we are focused on developing optimal parameters to make the motion triangle *ABC* which existed in the whole cycle avoid some extreme situations and make the tail fin's behavior mimic real fish's caudal fin. In order to simplify the problem, the influence of friction and gravity is supposed to be ignored.

### 4.1. Rod Length Relation of the Planar Linkage

Based on the motion triangle *ABC*, the side length relation could be written as

(12) l1+ΔS≥l2,l2+ΔS≥l1,l1+l2≥ΔS.

Simultaneous (12) and (8), the rod length must be satisfied with the following equation:

(13) $$\begin{array}{c} l_{1}=l_{2}\geq \frac{{\left|\Delta S\right|}_{\max}}{2}. \end{array}$$

Therefore, the swing angle *θ*_1_ in (9) could be simplified as

(14) $$\begin{array}{c} \theta_{1}=-\arcsin\left(\;\frac{D\sin\left(\phi /2\right)\sin\left(\phi /2+\omega t\right)}{2l_{1}}\;\right). \end{array}$$

**Figure 5:**
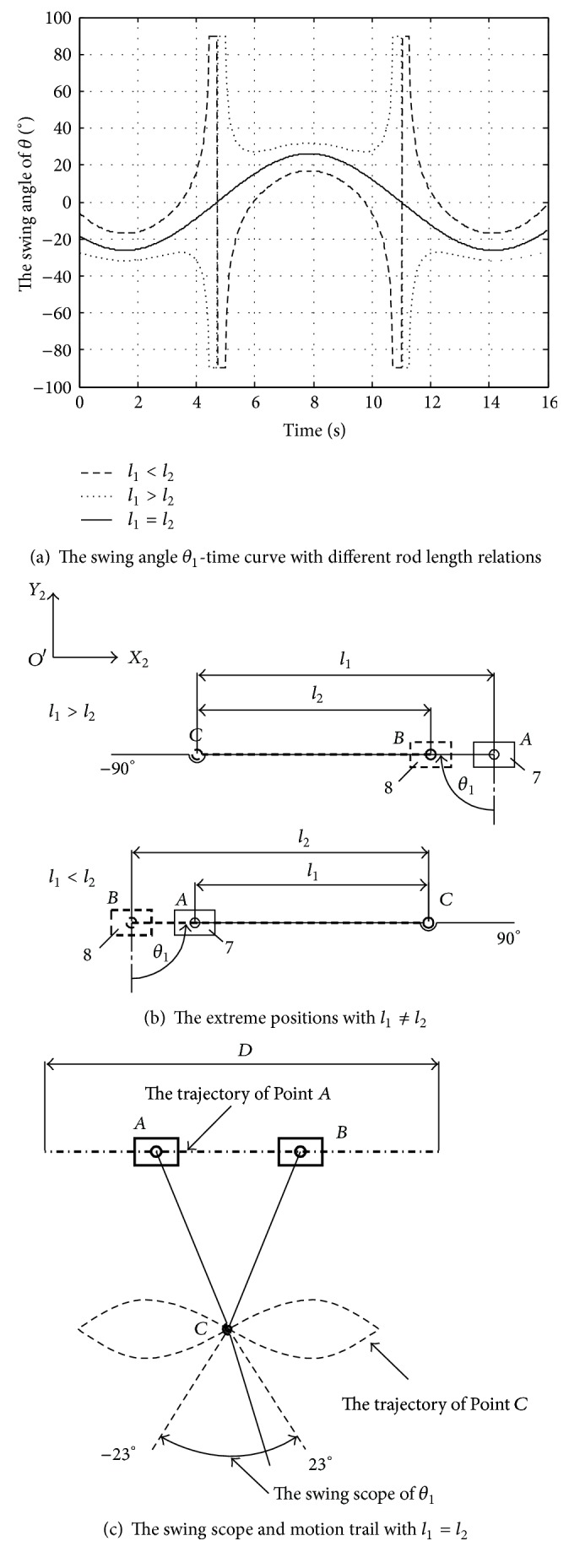
The rod length principle.

Based on (14), the length principle with *ω* = 0.5 rad/s, *ϕ* = 0.5*π*, and *D* = 100 mm is verified in MATLAB, as shown in Figure 5(a).

From Figure 5(a), when the rod lengths of the tail link 6 and the link 5 are unequal, the swing angle *θ*_1_ of the tail link 6 would vary irregularly and discontinuously, two sudden change points with peaking at 90° and −90° as shown in Figure 5(b). The two extreme positions at horizontal direction would cause destruction of the mechanism in its weak link joint. Only when *l*_1_ = *l*_2_, as shown in Figure 5(c), the swing angle *θ*_1_ could vary regularly and smoothly with a sinusoidal motion in the whole cycle, and the trajectories of Point *C* are two sine waves which are symmetric about *O*′*X*_2_ axis.

### 4.2. The Relation of Structural Parameters

The rod length of the tail link is also decided by stroke value in reciprocating, swing amplitude *θ*_max_ of the tail link, and phase difference *ϕ* with *l*_1_ = *l*_2_, and the rod length *l*_1_ can be described by

(15) $$\begin{array}{c} l_{1}=\frac{{\left|\Delta S\right|}_{\max}}{2\sin\theta_{\max}}=\left|\;\frac{D\sin\left(\phi /2\right)}{4\sin\theta_{\max}}\;\right|. \end{array}$$

**Figure 6:**
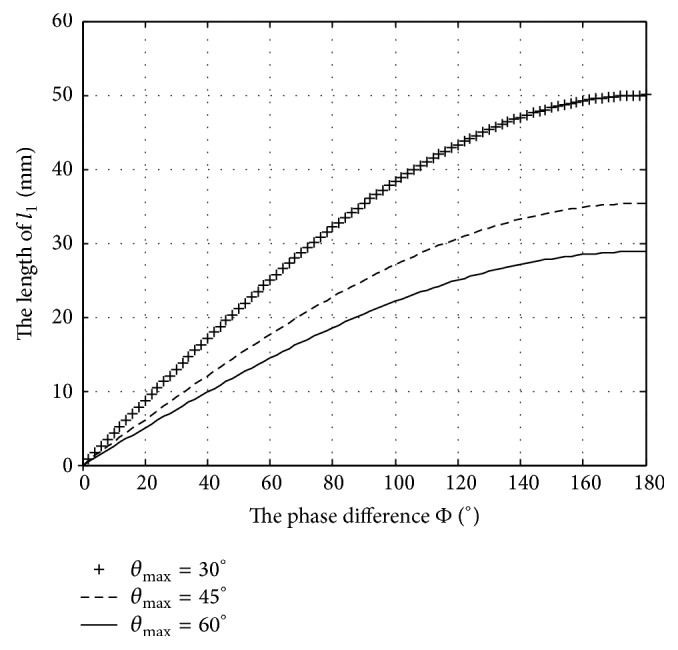
The rod length of the tail link varies with the phase difference.

**Figure 7:**
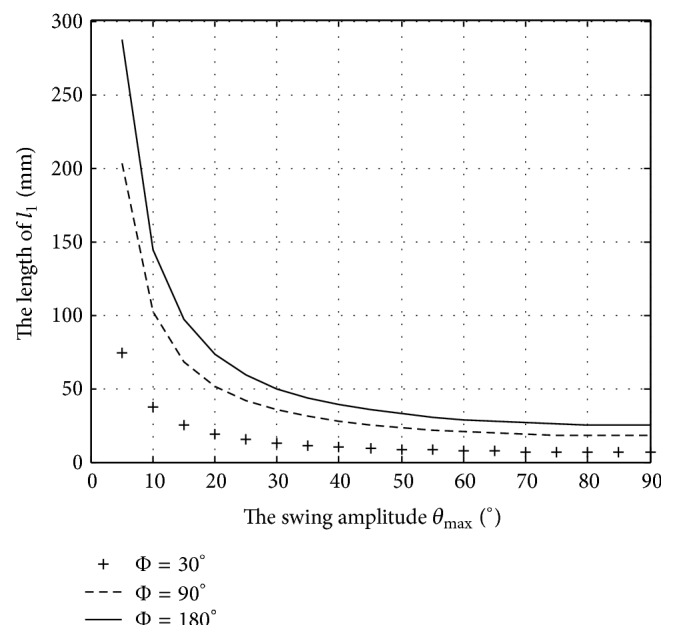
The rod length of the tail link varies with the swing amplitude.

With parameters *D* = 100 mm and swing amplitude *θ*_max_ = 30°, 60°, and 90°, respectively, the relation between the rod length *l*_1_ and phase difference *ϕ* is shown in Figure 6. With parameters *D* = 100 mm and phase difference *ϕ* = 30°, 60°, and 90°, respectively, the relationship between the rod length *l*_1_ and swing amplitude *θ*_max_ is shown in Figure 7.

From Figure 6, if the swing amplitude *θ*_max_ is fixed, the rod length can be adjusted to be shorter by decreasing the phase difference to gain more compact structure, and the rod length would peak when phase difference reaches 180°. The smaller the swing amplitude becomes, the faster the growth rate of the rod length would tend to grow. From Figure 7, the rod length could be adjusted to be shorter by decreasing the swing amplitude *θ*_max_ with a fixed phase difference, and the rod length will be the minimal length when the swing amplitude *θ*_max_ reaches 90°.

**Figure 8:**
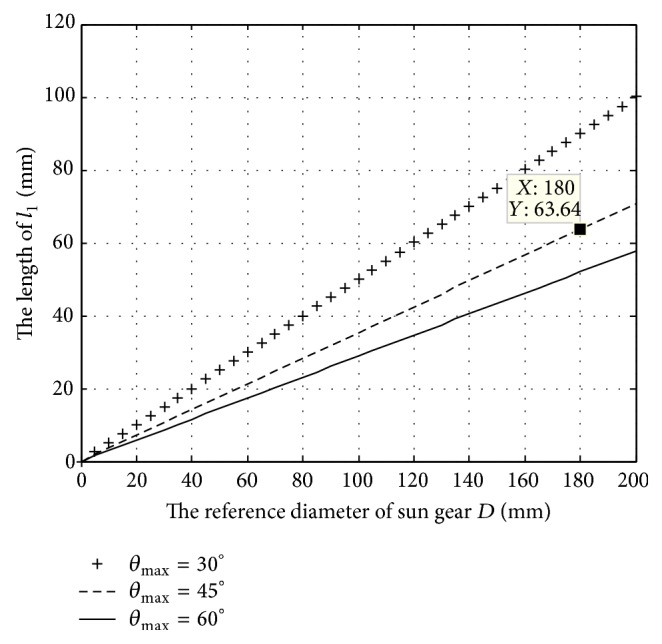
The rod length varies with the reference diameter of the sun gear.

Except for the phase difference and the swing amplitude, the rod length *l*_1_ is still determined by the reference diameter of the sun gear. With *ϕ* = *π* and *θ*_max_ = 30°, 45°, and 60°, respectively, the rod length varies with the reference diameter of the sun gear shown in Figure 8. The rod length increases linearly with the reference diameter of the sun gear, and the increase of the rod length would slow down with increasing the swing amplitude.

## 5. Design for an Application Example of the Driving System

### 5.1. A Sample of the Tail Driving System

A sample of the tail driving system was designed with parameters *D* = 180 mm, *ϕ* = *π*, *ω* = 1 rad/s, and *l*_1_ = *l*_2_ = 64 mm. The motion equation of the tail fin link could be specific as

(16) θ1=−arcsin⁡2sin⁡π2+t,SA=0.09cos⁡t.

### 5.2. Parameters of the Live Fish

**Figure 9:**
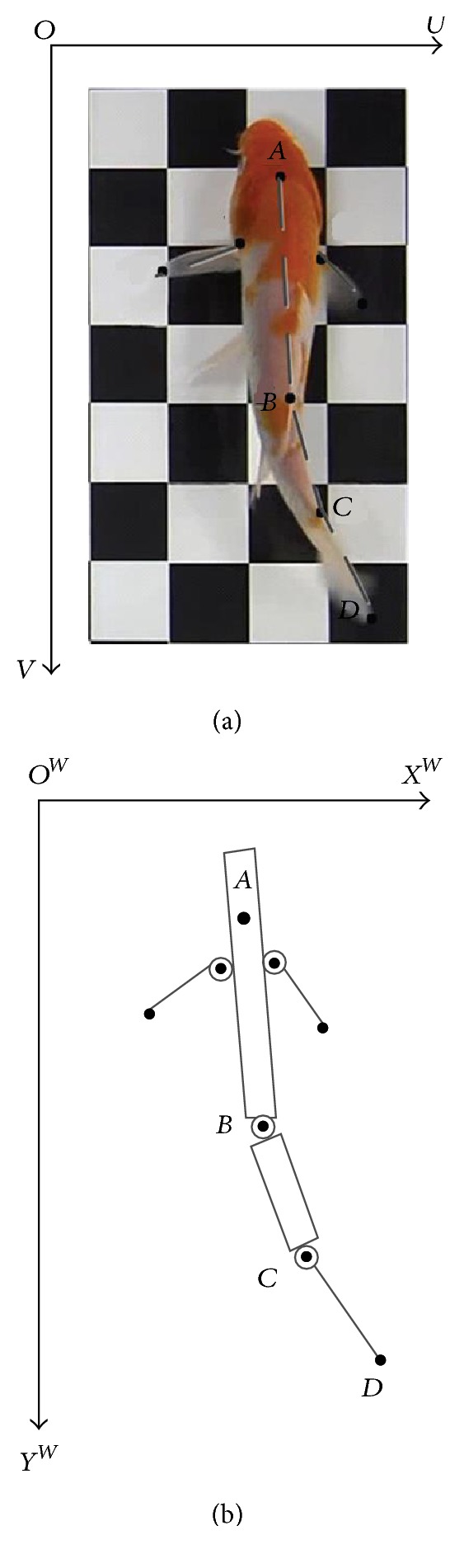
The Carp swimming image of eight feature points [12].

**Figure 10:**
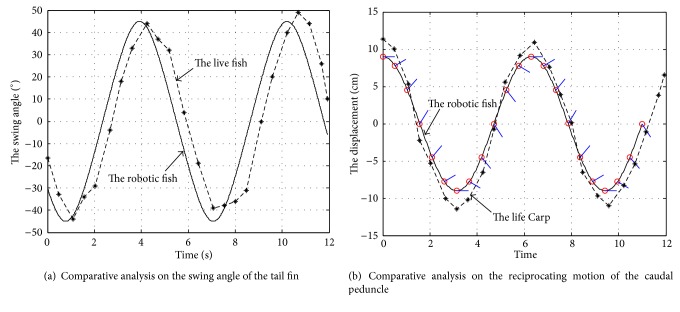
Comparative analysis of the driving system and a live fish.

The researchers of National University of Singapore have observed a real Carp with a length of 190 mm by PIV [12]. In their works, they selected four feature points for estimating Carp's joint angles but focused on kinematics studies of Point *C* located on Carp's peduncle and Point *D* located on its tail end, as shown in Figure 9. The motion of Point *C* and the swing angle of Point *D* were collected by videotaping the motion of fish at 60 frames per second using a video recording system. From the empirical observation in “Cruise” swimming, Carp continued to move in a nearly straight line at a constant speed, and the caudal fin flapped periodically. The trajectory of the caudal fin's swing angle is shown in Figure 10(a) with dotted line, and the trajectory of caudal peduncle's reciprocating is shown in Figure 10(b) with dotted line.

### 5.3. Simulation and Comparative Study

From Figure 10, the symmetry harmonic movement of the driving system coincided with the live fish. As Figures 10(a) and 10(b) have shown, the swing angle of the caudal fin in our designed driving system is similar to the live fish, and the swing amplitude could reach 45°. The reciprocating motion of the caudal peduncle is also similar to the live fish, and the stroke could reach 18 cm. When the caudal peduncle is situated at the limiting positions (*S*_*A*_ = 9 cm or −9 cm), the swing angle of the tail link will be zero. When the caudal peduncle is in the balance position with *S*_*A*_ = 0, the swing angle of tail fin reaches the maximum: that is, *θ*_1_ = 45°. Above all, the phase difference between the motion of caudal peduncle and tail fin's swing is about 90° and the phase difference of two planetary gears is 180°; that is, the phase difference of the actual output from the mechanism equals the half of input phase difference.

## 6. Conclusion

Based on linear hypocycloid, the driving system composed of a planetary gear train and a linkage has been developed for the tail transmission of two-joint bionic fish. The model and kinematics analysis of tail driving system were deduced by vector graphic method in this paper. The optimization of structure sizes was comprehensively studied to improve the kinematics performance. The simulation and comparative study on a bionic fish with a live fish were conducted so as to testify the feasibility of the driving system.

The results of structure size optimization show that the two rod lengths are equal to realize the tail oscillating continuously in cycle. In addition, the diameter of the sun gear *D*, the phase difference *ϕ* of two planetary gears, and the tail swing amplitude *θ*_max_ together affect the rod length *l*_1_. The rod length will increase with the growth of *D* and *ϕ* but decrease with the growth of *θ*_max_.

With the optimized structure, simulation and comparative study with a sample size (*D* = 180 mm, *d* = 90 mm, *ϕ* = *π*, *ω* = 1 rad/s, and *l*_1_ = 64 mm) in MATLAB have been conducted with experimental results of a live Carp to verify the feasibility of the driving system. These studies will work for future experiment study and the development of the mechanism design in underwater propulsion.
